# School refusal behavior in children and adolescents: a five-year narrative review of clinical significance and psychopathological profiles

**DOI:** 10.1186/s13052-024-01667-0

**Published:** 2024-05-30

**Authors:** Cristina Di Vincenzo, Maria Pontillo, Domenica Bellantoni, Michelangelo Di Luzio, Maria Rosaria Lala, Marianna Villa, Francesco Demaria, Stefano Vicari

**Affiliations:** 1 Child & Adolescent Neuropsychiatry Unit, Bambino Gesù Children’s Hospital, IRCCS, Piazza Sant’Onofrio 4, Rome, 00165 Italy; 2 Department of Life Sciences and Public Health, University Cattolica del Sacro Cuore, Rome, 00168 Italy

**Keywords:** School phobia, Neurodevelopmental disorder, Psychiatric disorder, Bullying, School-based interventions

## Abstract

The aim of the study was to explore the clinical significance of school refusal behavior, its negative impact on psychological well-being of children and adolescents and its relationship with the most common psychopathological conditions during childhood and adolescence (e.g. neurodevelopmental disorders, psychiatric disorders). School refusal behavior refers to a distressing condition experienced by children and adolescents that compromise regular school attendance and determine negative consequences on mental health and adaptive functioning. A narrative review of the literature published between January 2019 and March 2023 was conducted. Ten studies (*n* = 10) were included from a literature search of the electronic databases PubMed, CINAHL, PsycInfo, MedLine, and Cochrane Library. The results indicate that school refusal is highly present in neurodevelopmental disorders such as autism and attention-deficit/hyperactivity disorder due to the presence of behavioral problems and deficits in communication skills. As for psychiatric disorders, school refusal appears to be highly common in anxiety disorders, depressive disorders, and somatic symptoms. We also found that school refusal behavior may be associated with various emotional and behavioral conditions that act as risk factors. Especially, but are not limited to, it may be associated with a diminished self-concept, exposure to cyberbullying, specific affective profiles and excessive technology usage. Our results indicate that school refusal is a condition with many clinical facets. It can be attributed to both vulnerability factors, both temperamental and relational, and to various psychopathological conditions that differ significantly from each other, such as neurodevelopmental disorders and psychiatric disorders. Recognizing these aspects can improve the implementation of patient-tailored therapeutic interventions that are consequently more likely to produce effective outcomes. The therapeutic intervention should facilitate the recognition of cognitive biases regarding school as a threatening environment, while regulating negative emotions associated with school attendance. Additionally, therapeutic intervention programs linked to social skill training and problem-solving training, conducted directly within the school setting, can enhance children’s abilities to cope with academic performance and social relationships, ultimately preventing school refusal.

## Introduction

In 1941, Johnson first described “school phobia” as a childhood emotional disturbance characterized by pronounced anxiety, leading to excessive school absences [1, 2]. Subsequently, in 1960, Hersov coined the term “school refusal” to refer to this condition [3]. School refusal behavior is a specific type of school attendance problem (SAP) characterized by a complex interplay of emotional distress, parental awareness, absence of severe antisocial behavior, and parental commitment to fostering their child’s consistent school attendance, thus distinguishing it as a distinct category within the realm of school attendance problems. Firstly, it involves a young individual who displays a marked reluctance or refusal to attend school. This reluctance is invariably accompanied by emotional distress that is symptomatic of an aversion to school attendance. This emotional distress may manifest as excessive fearfulness, unhappiness, or unexplained physical symptoms. Alternatively, it could take the form of chronic emotional distress, such as a depressive affect or sleep disturbances, which, while not necessarily resulting in complete absence, significantly impairs regular attendance. Furthermore, the young person does not try to hide school absences from their parents (e.g., they are at home and the parents are aware of this).

## Prevalence and diagnosis

It’s noteworthy that apart from the resistance shown toward attending school, young individuals with school refusal behavior typically do not exhibit severe antisocial behaviors, except for resistance to parental efforts to get them to attend school. Lastly, a critical aspect of school refusal behavior involves the efforts made by parents to ensure their child’s regular attendance at school [4]. Approximately 2–5% of all school-aged children and adolescents experience school refusal behavior. The occurrence is comparable between males and females. While school refusal behavior occurs at any ages, it is more prevalent in children aged 5 to 6 and those between 10 and 11 years of age [2]. School refusal behavior can be characterized as a symptom that can be associated with several other unspecific clinical signs. The most frequently reported symptoms include abdominal pain, headache, nausea, vomiting, muscular or joint pain, diarrhea, dizziness, fatigue, and palpitation [5] with negative impact on the mental health, educational success and social functioning of children and adolescents. Finally, school refusal behavior is directly linked with high degree of parental psychological distress [6].

Despite this, school refusal behavior is a condition not included in a Diagnostic and Statistical Manual of Mental Disorders (DSM–5) diagnosis. To date, it can be detected and diagnosed by Berg’s criteria [7] reviewed by Heyne et al. [6]. Specifically, these criteria are: [1] less than 80% attendance during the past two school weeks (excluding legitimate absences); [2] presence of a psychiatric disorder identified by DSM anxiety disorder (excluding obsessive-compulsive disorder and posttraumatic stress disorder); [3] parents could account for their child’s where abouts on days of absence; [4] no current conduct disorder (less serious behavioral disturbance in the form of oppositional defiant disorder was permitted); [5] current expressed parental commitment for their child to achieve regular school attendance (i.e., full attendance except for legitime absences) [8].

School refusal behavior should not be only attributed to school problems; instead, it should be considered in a larger set of processes [8]. Recent studies have attempted to explain school refusal behavior by examining vulnerability factors, as well as individual, environmental and family risk factors [9]. Regarding individual risk factors, previous studies has found associations between school refusal behavior and anxiety, as well as a history of learning disabilities [10–12]. In terms of environmental factors, abuse, child maltreatment and bullying are prevalent in children and adolescents with school refusal behavior [13]. Additionally, recent studies have found links between school refusal behavior and family-related factors such as separation, household conflicts, and parental psychiatric illness [14].

In addition, school refusal behavior is the core symptom in different psychiatric disorders. The most common are social anxiety disorder, generalized anxiety disorder, specific phobia, major depression, oppositional defiant disorder, post-traumatic stress disorder, adjustment disorder, among others [2]. Previous research has also found that the onset of school refusal behavior occurs at a younger age in children with multiple co-occurring developmental disabilities. Specifically, children with Attention Deficit and Hyperactivity Disorder (ADHD), Autism Spectrum Disorder (ASD), and Intellectual Disability (ID) were more likely to experience chronic school absenteeism compared with neurotypical children [1, 15, 16]. Based on this, the objective of our narrative review is to examine the clinical significance of school refusal behavior, its negative impact on psychological well-being of children and adolescents and its relationship with the most common psychopathological conditions during childhood and adolescence. More in detail, our aim was to deepen the school refusal behavior in children and adolescents with neurodevelopmental disorders and those with psychiatric disorders (e.g., anxiety and depressive symptoms), exploring clinical profile and risk factors. We propose that school refusal behavior has different clinical meanings depending on underlying psychopathological frameworks and different vulnerability factors (e.g., temperamental traits, bullying, etc.), and that the knowledge of these factors can facilitate the development of specific, differentiated, and therefore more effective interventions.

## Methods

The present study comprised a narrative review of the literature published between January 2019 and March 2023, according to the Preferred Reporting Items for Systematic Reviews and Meta-Analyses (PRISMA) guidelines.

### Search strategy and selection criteria

All included studies were obtained from a literature search of the electronic databases PubMed, CINAHL, PsycInfo, MedLine, and Cochrane Library. Expression used in the search included: (“School refusal behavior”) AND (“Anxiety disorder” OR “Neurodevelopmental disorder” OR “Psychopathological disorders”) AND (“children” OR “adolescents”). Finally, the references of all articles entered the review were manually searched.

### Selection criteria

The search was focused on the children and adolescents.

The inclusion criteria were:

1. Original Research Articles.
2. Observational articles (cross-sectional study, retrospective study).
3. Experimental study.
4. Studies reporting the percentage or prevalence frequency of school withdrawal in neurodevelopmental disorders and psychiatric disorders.

The exclusion criteria were:

1. Studies unrelated to the objective of the study.
2. Article format (e.g., review, comments, letters).
3. Sample characteristics: only specific population included.
4. Not included quantitative and standardized data (case series study).
5. Article for validation instrument.

### Selection process of study

Following the establishment of the search strategy for each database, overlapping studies across various databases were eliminated. In the subsequent phase, the titles and abstracts of the studies underwent a review, and studies deemed unrelated were excluded. Following this, the full texts of the remaining articles were meticulously examined in accordance with predetermined inclusion and exclusion criteria, leading to the exclusion of irrelevant studies. Ultimately, articles meeting all inclusion criteria proceeded to undergo qualitative evaluation. No language restrictions or study design restrictions were applied.

### Selection procedure

The reference lists for articles that meet the inclusion criteria were examined. The search algorithm retrieved a total of 317 articles (PubMed: 204 results; Psychinfo: 5 results; MedLine 0 results; Cochrane library: 0 results; Cinahl: 8 results), of which 298 were excluded prior to screening. Of the 19 records screened, 10 referred to eligible studies, and the remaining 9 were excluded for the reasons listed in Table 1.

**Table 1:** Excluded studies and the reasons for their exclusion

Reason for Exclusion	Study Name
Article format (e.g., review)	[3, 5, 17]
Sample characteristics: only specific population included	[18, 19]
Not included quantitative and standardized data (e.g. case series study)	[20]
Article for validation instrument	[21]

### Risk of bias assessment

To ensure the reliability and quality of the review, and to thoroughly analyze the outcomes of the selected studies, a bias analysis was conducted. This analysis followed the guidelines and criteria outlined by the Agency for Health Care Research and Quality [22]. Each study underwent bias assessment according to predetermined criteria, encompassing selection bias, performance bias, detection bias, attrition bias, and reporting bias. Subsequently, we assessed the risk of bias for each study, ranging from medium to low. Variability among the included studies was mitigated by strict inclusion criteria. Specifically, all chosen articles were original research articles focusing on a pediatric population aged 4 to 18 years. These articles included both observational and experimental studies to ensure greater internal validity and broader generalizability. However, limitations regarding participant selection and sampling methods may restrict the generalizability of the results, as participants were not consistently drawn from clinical populations. Of the eight included studies, the majority utilized standardized and scientifically validated assessment tools, and employed both univariate and multivariate statistical methods. These methods enhance the reliability and generalizability of the findings. However, two studies employed a Likert scale and a checklist developed by the authors themselves, which are non-standardized instruments. The use of such instruments may hinder the generalizability of the results. Furthermore, a potential limitation affecting the generalizability of the results is that the studies primarily focused on populations from Europe and North America, rather than being cross-cultural in nature.

**Fig. 1:**
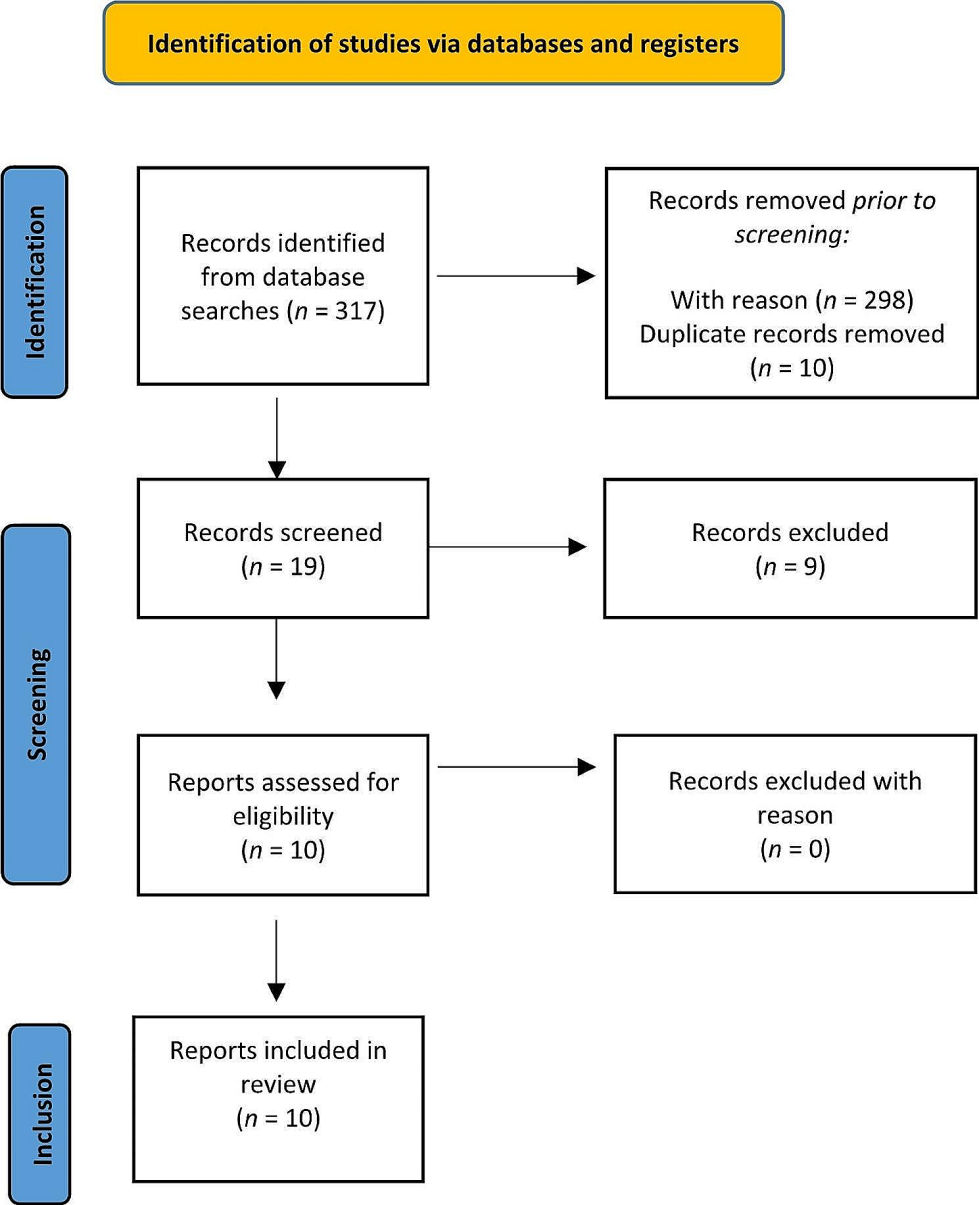
Flow chart of literature review

In terms of evidence-based medicine, the quality of the included studies was moderate. Figure 1 presents a detailed flow diagram of the study selection process.

## Results

Due to the low number and heterogeneity of the included studies, a narrative synthesis was conducted to describe, organize, explore, and interpret their findings while examining their methodological adequacy. Table 2 describes the methodologies and results of the ten studies that we included.

**Table 2:** Methodologies and results of the investigated studies

Study	Sample	Method(s)	Measures	Results
Gonzalves et al. [23]	Sample: 1315 Spanish students (range age: 12–18)	Experimental study	SRAS-R SDQ-II-Short Form	Four different School Refusal Behaviour profiles: Moderately High School Refusal Behaviour(n 489, (37.2%)), Moderately Low High School Refusal Behaviour (n 433 (32.9%)) Mixed School Refusal Behaviour (n 177, (13.5%), Non-School Refusal Behaviour (n 216 - (16.4%). Mixed School Refusal Behaviour group was the most maladaptive profile and revealed the lowest mean scores on self-concept.
Delgado et al. [26]	Sample: 1,102 Spanish high school students aged 12–18 years (mean age 14.30; SD 1.71).	Experimental study	SRAS-R	Results showed 3 behaviour profiles: (1) SRB by negative reinforcements (419–38.02%) (2) SRB by positive reinforcements (389 -35.29%) (3) non-SRB students (267- 24.22%). The first group showed higher rates than the others in victimization, aggression, both, and observation of cyberbullying.
Gonzalvez et al. [24]	Sample: 1,816 Spanish adolescents (range age: 15–18 years)	Experimental study	PANAS-C-SF SRAS-R-C	5 affective profiles: a) low affective profile (*n* = 40; 2.2%), b)self-fulfilling profile (*n* = 899; 49.5%),c) low positive affect profile (*n* = 698; 38.4%), d) self-destructive profile (*n* = 86; 4.7%), and e) high affective profile (*n* = 93; 5.1%). Statistically significant differences were found among profiles in the four conditions of SRAS-R-C (*p* < 0.001).
Fujita et al. [25]	Sample: 227 students, aged 10–18 years, with any psychiatric disorder (except moderate-to-severe or profound intellectual disability), showing SRB, defined as at least 30 days of absence from classes, and Problematic Internet Use (PIU)	Observational cross-sectional analytical study	IAT QCD GAD-7 PHQ9	46/112 (41.1%) students with SRB exhibited PIU, with an IAT score > 50. They showed lower CQD scores in each part of day except at night (p range 0,5 − 0,05) and significant higher scores in PHQ9 and GAD-7 tests and more frequently diagnosed with mood disorders.
Ellen Kathrine Munkhaugen et al. [27]	Sample: 62 individuals (age range 9–16 y- mean age 12.3) whit ASD, without intellectual disability, divided into 2 groups: ASD plus SRB (n 33- 53.2%) and ASD without SRB (n 29)	Experimental study	BRIEF SRS CBCL	Significant difference between the two groups in: social functioning (*p* = 0.002) measured by SRB, in executive functions (*p* = 0.002) measured by BRIEF, in emotional and behavioural problems (*p* = 0.001) measured by CBCL.
Vicki Bitsika et al. [28]	Sample: 67 mothers and their sons (age range 7–18 ), with ASD, bullying experience and school refusal (SR)	Experimental study	CASI-4	Boys with SR had significantly higher GAD and MDD than boys without SR (*p* = 0.13) and the frequency of being bullied made a significant contribution to emerging SR (*p* = 0.004)
Abbey j. McClemont et al. [29]	Sample: 97 parents with 154 children (age range 4–16 y) aged at least 18 with diagnosis of ASD (*n* = 36), ADHD (*n* = 16), ASD + ADHD (*n* = 31), other diagnosis (i.e., anxiety disorders, mood disorders, disruptive behaviour disorders, learning disorders, language/communication disorders, sensory/auditory processing disorders; *n* = 15), and no diagnosis (*n* = 56).	Brief Report	LIKERT scale	35% of parents state that their child has never refused school because of bullying. A significant difference between groups in lifetime school refusal due to bullying (*p* < 0.001). Children with ASD + ADHD were more likely to have ever refused school because of bullying (68%) than children with ASD (28%) or no diagnosis (18%).
Carpentieri et al. [30]	Sample: 103 adolescent with a mean age of 16.2 (SD ± 1.14), divided into two group: with school refusal (SRa) *n* = 28 and not school refusal (non-SRa) *n* = 75	Experimental study	HAM-A HAM-D YMRS GAF GFSS GFRS SWAP-200-A	SRa showed higher anxious and depressive symptoms (HAM-A *p* = 0.036; HAM-D *p* = 0.031), lower level of global functioning (GAF < *p* < 0.001, GAF_past year *p* = 0.025), lower levels of social and role functioning (GFSS *p* = 0.003, GFRS *p* = 0.002), higher rates of Schizoid (*p* = 0.046) and Schizotypal (*p* = 0.034) personality disorders, lower Health Functioning (*p* = 0.001) and the Q Health Index (*p* = 0.002).
Al Keilani and Delvenne [8]	Sample: 71 patients (age range: 8–16 years) from the Child and Adolescent Psychiatric Department of Queen Fabiola Children’s University Hospital, with an anxious school refusal behaviour (ASR)	Retrospective study	**A checklist** including: patient’s gender, status, age of onset of school refusal, age of assessment, duration of school refusal, associated events, life events, use of psychotropic medication, family psychiatric history and family composition, individual psychiatric history	Significant sex difference: 70.42% of male (*p* < 0.0003) with ASR. Risk factors: family separation (56.3%), conflict at home (27%), contact rupture with father (25.3%), maternal psychiatric illness (45.07%), paternal psychiatric illness (28.2%), academic difficulties (36.6%) and change school or moving home (19.7%). Concerning psychopathological diagnosis, anxiety (39.4%) and mood disorder (32.4%).
Xavier Benarous et al. [31]	Sample: 191 adolescents aged 12–18 years (M = 15.0, 44% boys)	Retrospective chart review study	C-GAF CGI-S DEP-ADO	7% with SW/SR (*n* = 83) met HKM criteria (*n* = 14, M = 14.3, 64% boys), accounting for one in six adolescents with SW/SR. No significantly differ from the other forms or SW/SR in terms of demographic factors, academic performance or psychosocial factors. SW/SR and HKM + patients had higher rates of anxiety disorders (Odd Ratio, OR = 35.2) and lower rates of disruptive behavioural disorders (OR = 0.03). None of the HKM + reported use of illicit drugs, alcohol, compared to 25% of youths with other SW/SR.

Behaviour Inventory of Executive Function (BRIEF), Child and Adolescent Symptom Inventory-revision 4 (CASI-4), Children-Global Assessment of Functioning scale (C-GAF), Clinical Global Impressions-Severity scale (CGI-S), Child Behaviour Checklist (CBCL), General Anxiety Disorder-7(GAD-7), Global Assessment of Functioning (GAF), Global Functioning Social Scale (GFSS), Global Functioning Role Scale (GFRS), Hamilton Rating Scale for Anxiety (HAM-A), Hamilton Rating Scale for Depression (HAM-D), Internet Use with Internet Addiction Test (IAT), Patient Health Questionnaire − 9 (PHQ9), Positive and Negative Affect Schedule Short Form (PANAS-C-SF), Questionnaire-Children with Difficulties (QCD), School Refusal Assessment Scale-Revised for Children (SRAS-R-C), Self-Description Questionnaire (SDQ-II-Short Form), Shelder-Westen Assessment Procedure for Adolescents (SWAP-200-A), Social Responsiveness Scale (SRS), Young Mania Rating Scale (YMRS)

### School refusal behavior: analysis of risk factors and impacts on student well-being

#### The role of self-concept

Gonzalves et al. [23] identified various profiles of school refusal behavior and analyzed whether there were significant differences in the scores of eleven dimensions of self-concept construct (Physical appearance, Physical abilities, Parent relations, Same-sex relations, Opposite-sex relations, Honesty, Emotional stability, Self-esteem, Verbal, Math, and General school) among these different profiles. In this study, self- concept refers to the set of perceptions that form the image that a person has of themself and in its configuration, both cognitive and social aspects come into play. The study comprised 1315 Spanish students, ranging in age from 12 to 18 years. School refusal behavior was assessed using the School Refusal Assessment Scale-Revised (SRAS-R), a self-report measure consisting of 24 items. It is scored on a 7-point Likert scale (0 = never; 6 = always) and evaluates four conditions contributing to the maintenance of school refusal behavior: avoidance of school-related stimuli that provoke a sense of general negative affectivity (ANE), escape from aversive social and/or evaluative situations at school (ESE), pursuit of attention from significant others (PA), and pursuit of tangible reinforcement outside of the school setting (PTR). Of those four factors, the levels of reliability was ranged from 0.70 (Factor I) to 0.87 (Factor III). The coefficients of internal consistency (Cronbach’s alpha) obtained in the present study ranged from 0.71 (Factor IV) to 0.84 (Factor II). Self-concept was evaluated using the Self-Description Questionnaire (SDQ-II-Short Form), a self-report measure with 51 items scored on a 6-point Likert scale (1 = false; 6 = true) with a coefficient of internal consistency (Cronbach’s alpha) ranged from 0.60 (Opposite-sex relations) to 0.77 (Physical appearance). The study employed latent class analysis (LCA) to categorize participants based on school refusal behavior scores, specifically across the four functional dimensions of the SRAS-R. LCA, known for its precision, was chosen over K-means clustering to address limitations. Model fit was rigorously assessed using the Bayesian information criterion (BIC) and Entropy values to determine the optimal number of latent classes. After identifying school refusal behavior profiles, the study proceeded to analyze inter-class differences in scores on seven dimensions of self-concept using analysis of variance (ANOVA). Post hoc tests with the Bonferroni method were conducted to further explore specific group differences, and effect sizes (Cohen’s d index) were calculated to quantify the observed differences. The interpretation of Cohen’s d values followed established guidelines for effect magnitude. The results of the study revealed four distinct School Refusal Behavior profiles: Moderately High School Refusal Behavior, which included 489 students (37.2%) characterized by moderate scores in all SRAS-R dimensions; Moderately Low High School Refusal Behavior, which included 433 students (32.9%) characterized by a low level of school refusal behavior primarily driven by the pursuit of attention from significant others (the third factor of SRAS-R) and moderately low levels in the remaining SRAS-R dimensions; Mixed School Refusal Behavior, including 177 students (13.5%) with high levels of school refusal behavior driven by avoidance of school-related stimuli that provoke a sense of general negative affectivity, escape from aversive social and/or evaluative situations at school, and pursuit of attention from significant others (the first three dimensions of SRAS-R); Non-School Refusal Behavior, which included 216 students (16.4%) with low scores of school refusal behavior across all dimensions investigated by SRAS-R. Furthermore, the Mixed School Refusal Behavior group exhibited the lowest scores in self-concept, while the Non-School Refusal and Moderately Low School Refusal Behavior groups demonstrated the highest scores in all dimensions of self-concept. The study highlights that high levels of school refusal behavior in adolescents are associated with the avoidance of school-related stimuli that evoke a general sense of negative affectivity. This behavior includes escaping from aversive social and evaluative situations at school, as well as seeking attention from significant others. Additionally, these adolescents are at the greatest risk of having a negative self-perception.

### Psychological factors: affective profile and school refusal behavior

In a subsequent study, Gonzavez et al. [24] investigated different affective profiles and analyzed the differences between these profiles based on school refusal behavior. The study involved 1,816 Spanish adolescents (range age: 15–18 years). Affect was assessed using the Positive and Negative Affect Schedule Short Form (PANAS-C-SF), a 10-item self-report measure rated on a 5-point Likert scale (ranging from 1 = very slightly or never to 5 = very much). This scale consists of two subscales, measuring the positive affective dimensions (joyful, lively, happy, energetic, and proud) and the negative affective dimensions (depressed, angry, fearful/scared, afraid, and sad) of affectivity. The two subscales showed appropriate internal consistency values in the original study (positive affect.86; negative affect.82) and in this study (positive affect.82; negative affect.71). The study implemented a variety of statistical analyses, including both univariate and multivariate approaches, to explore the relationships between affectivity and school refusal behavior. Initially, Pearson’s product–moment correlation coefficient was employed to assess correlations between positive and negative affect and different conditions of school refusal behavior. Subsequently, Latent Profile Analysis (LPA) was conducted to identify cluster solutions for the two-factor conceptualization of affectivity. The study rigorously evaluated the fit of various LPA models using multiple fit statistics criteria, ensuring the selection of the most adequate class solution. To test group differences, a multivariate analysis of variance (MANOVA) was utilized, considering the dimensions of school refusal behavior between the identified affective profiles. The effect sizes were calculated using the d index to provide a measure of the observed differences. Latent profile analysis revealed five affective profiles: low affective profile (*n* = 40; 2.2% of the sample), self-fulfilling profile (*n* = 899; 49.5% of the sample), low positive affect profile (*n* = 698; 38.4% of the sample), self-destructive profile (*n* = 86; 4.7% of the sample), and high affective profile (*n* = 93; 5.1% of the sample). The researchers then examined the influence of four motivating factors for school refusal behavior: avoidance of stimuli that provoke negative affectivity (F1); escape from aversive social and/or evaluative situations (F2); pursuit of attention from significant others (F3); pursuit of tangible reinforcement outside of school (F4). These factors were assessed using the Spanish version of the School Refusal Assessment Scale-Revised for Children (SRAS-R-C), an 18-item self-report measure rated on a 7-point Likert scale (ranging from 0 = never to 6 = always). In this study, the coefficients of internal consistency were 0.64, 0.73, 0.78, and 0.56 for factors 1(ANE), 2(ESE), 3(PA), and 4(PTR), respectively. Statistically significant differences were observed among profiles in the four conditions of SRAS-R-C (*p* < 0.001; η2p = 0.03). Specifically, the “self-destructive profile” exhibited the highest average scores in the first three factors of the SRAS-R-C, while the “high affective profile” had the highest average scores in the fourth factor. These findings highlighted that the “self-destructive profile” represented the most maladaptive affective profile in terms of school refusal behavior. These findings underscore that the self-destructive profile is more related with school refusal behavior. Specifically, adolescents exhibiting traits such as fear, anger, nervousness, lack of interest, guilt, shame, and heightened temperamental sensitivity to negative stimuli are more prone to encountering difficulties in attending school, particularly in situations that elicit discomfort, anxiety, and/or depression.

### Problematic internet use and school refusal behavior

Fujita et al. [25]investigated the relationship between daily difficulties and Problematic Internet Use (PIU) in adolescents with School Refusal Behavior (SRB). Their specific objectives included examining differences in daily burdens between adolescents with PIU and those without PIU, as well as assessing the impact of depressive and anxiety symptoms on daily burdens among adolescents with PIU. They utilized the Internet Addiction Test (IAT), a self-report questionnaire that scores between 0 (minimum) and 100 (maximum), to analyze internet use and compare it between individuals with and without PIU. The sample comprised 227 students who were enrolled and exhibiting SRB. Daily difficulties were assessed using the Questionnaire-Children with Difficulties (QCD), administered by parents. Anxiety levels were quantified using the General Anxiety Disorder-7 (GAD-7), a 7-item self-report questionnaire scoring from 0 to 21, with scores of 11 or higher indicating significance and Cronbach’s alpha for the total score equal to 0.92. Depressive symptoms were assessed in students using the Patient Health Questionnaire-9 (PHQ-9), which includes 9 items with scores ranging from 0 to 27, and scores of 14 or more are considered significant; Cronbach’s alpha for the total score was 0.89. The study extensively utilized multivariate analyses, specifically linear regression models with adjustment for covariates, interaction terms, and multiple imputations to address missing data. These methods were employed to explore the associations between primary outcomes and Problematic Internet Use (PIU) while accounting for potential confounding variables such as age, sex, and various psychological diagnoses. Additionally, the authors conducted sensitivity analyses to assess the robustness of their findings, incorporating imputed responses and predictors under different assumptions about missing data patterns. Despite the absence of explicit mention in the abstracts, the study took careful measures to control for potential confounding factors and missing data issues. Among the 112 students with SRB who completed all the questionnaires, 46 (41.1%) exhibited PIU with an IAT score of 50 or higher. Compared to students without PIU, those with PIU exhibited significantly higher scores in the PHQ-9 and GAD-7 tests. No interactive effects were observed between PIU and depressive or anxious symptoms (p values < 0.5; 95%IC: -13,41 − 2,09). In summary, within the cohort of adolescents exhibiting school refusal behaviors, problematic internet use (PIU) appears to influence the daily difficulties assessed by parents. These difficulties induced by PIU were prevalent nearly all day and exhibited distinct characteristics when compared to symptoms of depression and anxiety.

### School refusal behavior and cyberbullying: is there a link?

Delgado et al. [26] analyzed the relationship between school refusal behavior and cyberbullying during the 2017–2018 academic year among 1,102 Spanish high school students aged 12–18 years (mean age 14.30; SD 1.71). The primary objective was to investigate differences in the School Refusal Assessment Scale-Revised (SRAS-R), a 24-item self-report questionnaire on a 7-point Likert scale, using Latent Class Analysis. In this study, the subscales of the questionnaire demonstrated an adequate reliability based on the Cronbach’s alpha values which were 0.77 for ANE, 0.75 for ESE, 0.80 for PA, and 0.78 for PTR. The study employs latent class analysis (LCA) to define profiles of School Refusal Behavior (SRB) based on the four functional conditions of the SARS-R. LCA is chosen as it is deemed most suitable for establishing profiles in large samples and addresses limitations found in other statistical techniques. The researchers use rigorous criteria, such as the Bayesian Information Criteria (BIC), Akaike Information Criterion (AIC), and Entropy, to determine the optimal number of classes that best represent the research data. This ensures a robust classification of subjects into distinct classes based on their SRB profiles. To assess the differences in cyberbullying (victimization, aggression, observation, and aggression-victimization) between the identified classes of SRB, the study employs ANOVAs, followed by post hoc Scheffé tests to identify specific groups with statistically significant differences. Additionally, the calculation of the d index (standardized mean difference) proposed by Cohen allows for the assessment of the magnitude or effect size of the observed differences. Three distinct profile groups were identified. The first group was labeled as “School Refusal Behavior (SRB) by negative reinforcements” (419 students or 38.02%), characterized by high levels of Avoidance of Negative Affectivity (ANE) and Escape from social and evaluative situations (ESE), and low levels of Pursuit of Attention (PA) and Pursuit of Tangible Reinforcement (PTR). The second group was labeled as “SRB by positive reinforcements” (389 students or 35.29%), marked by high levels of PA and PTR and low levels of ANA and ESE. The third profile consisted of “non-SRB students” (267 youths or 24.22%) with low scores across all four dimensions (ANA, ESE, PA, PTR). To assess differences in cyberbullying among the various SRB profiles, the Screening of Harassment Among Peers (SPH) questionnaire was administered. This self-report questionnaire consisted of 45 items with a Likert-like response scale (0 = never to 4 = always). Post hoc analysis revealed that students classified under the “SRB by negative reinforcements” profile exhibited higher rates of victimization, aggression, both victimization and aggression, and observation of cyberbullying compared to the other two groups. No differences were observed between the profiles of “SRB by positive reinforcements” and “non-SRB students. The study reveals that adolescents with a negative emotional component are more likely to exhibit school refusal behaviors to avoid negative emotions and social and academic evaluation situations. Moreover, these adolescents are more exposed to cyberbullying behaviors, both as victims and aggressor.

### School refusal behavior in children and adolescents with neurodevelopmental disorders

Autism spectrum disorder and school refusal behavior: the role of individual characteristic Ellen Kathrine Munkhaugen et al. [27] conducted a study to explore the individual characteristics associated with School refusal behavior (SRB) in students with autism spectrum disorder (ASD) comparing social executive functioning and emotional and behavioral problems between students with ASD and SRB and those with ASD without SRB. The study included a sample of 62 participants with ASD, without intellectual disability (range age: 9–16 years). Out of these participants, 33 exhibited ASD with SRB, while 29 had ASD without SRB. The school refusal behavior was evaluated with School Refusal Behavior questionnaire administered to parents. The executive functions were evaluated utilizing the Behavior Inventory of Executive Function (BRIEF), an 86-items parent-rated inventory employing a three-point Likert scale (1 = never a problem; 2 = sometimes a problem; 3 = often a problem). The BRIEF comprises eight subscales: Inhibit, Shift, Emotional, Control, Initiate, Working Memory, Plan/organize, Organization of materials and Monitor. Subsequently, the scores were transformed into a total score known as the Global Executive Composite (GEC). The severity of social impairment and ASD symptoms was evaluated using the Social Responsiveness Scale (SRS), a 64-item questionnaire rated on a four-point Likert scale (1 = not true; 2 = sometimes to; 3 = often true; 4 = almost always true). Additionally, emotional and behavioral problems were assessed through the Child Behavior Checklist (CBCL), a 112 items parent-self questionnaire rated on a three-point scale that consists of eight syndrome scales: Anxiety/Depressed; Withdrawn/Depressed, Somatic Complaints, Social Problems, Thought Problems, Attention Problems, Rule-Breaking Behaviors and Aggressive Behaviors. The study indeed employed a comprehensive set of statistical analyses to investigate the relationships and associations between different variables. The methods include univariate analyses, such as chi-square tests and t tests, as well as multivariate approaches like Multivariate Analysis of Variance (MANOVA), logistic regression, and stepwise logistic multiple regression. To control for multiple comparisons, the authors used Bonferroni correction within the subscales of SRS, BRIEF, and CBCL. Furthermore, effect sizes, specifically Cohen’s d, were calculated to assess the magnitude of the observed effects. The power calculations indicate that the sample size was adequate to detect medium to large effect sizes with sufficient statistical power. Overall, the study utilized a robust statistical approach to explore the relationships between variables while considering potential confounding factors. The study’s findings indicated significant difference between the two groups. There were differences in social functioning, as measured by the SRS, executive functions, as assessed by the BRIEF, and emotional and behavioral problems according to the CBCL. More specifically, differences were observed between students with and without SRB in the SRS Social Motivation subscale (*p* = 0.002; 95%IC: −17.1 to − 4.2; Cohen’s d = 0.8). Furthermore, the results demonstrated that the BRIEF GEC scores were higher in the student with ASD and SRB compared to those without SRB (*p* = 0.004; 95%IC: −12.8 to − 2.5; Cohen’s d = 0.7). Notably, two of eight subscales from the BRIEF showed differences between students with and without SRB: Initiate (*p* < 0.001; 95%IC: −17.3 to − 6.5; Cohen’s d = 1.1) and Plan/Organize (*p* < 0.001; 95%IC: −15.2 to − 4.5; Cohen’s d = 0.9). Finally, the CBCL results revealed higher problem scores in students with SRB compared to those without SRB (*p* = 0.001; 95%IC: −12.6 to − 2.5; 0.9 Cohen’s d). Differences were also identified between students with and without SRB across four of the eight CBCL subscales: Anxiety/Depression (*p* = 0.002; 95%IC: −12.7to − 2.1; Cohen’s d = 0.8), Withdrawn/Depressed (*p* < 0.001; 95%IC: −14.3to − 5.0; Cohen’s d = 1.2), Somatic Complaints (*p* = 0.005), and Thought Problems (*p* = 0.005; 95%IC: −11.0 to − 1.9; Cohen’s d = 0.7). These findings indicate that students with ASD and SRB, in contrast to those without SRB, exhibit lower social motivation, impaired abilities to initiate activities or tasks, difficulty generating ideas, responses, or problem-solving strategies, and display more symptoms of withdrawal and depression.

### Bullying and correlates of school refusal in autistic youth

Vicki Bitsika et al. [28] examined the role of bullying in emerging school refusal behavior among autistic youth, as well as potential correlates of School Refusal (SR). These potential correlates included the frequency of bullying experiences, the age of the autistic youth, parental assessments of the challenges faced by their autistic children, and the levels of anxiety and depression in autistic youth. The study included 67 mothers and their autistic sons (range age: 7–18). Data collection involved the use of a questionnaire package consisting of three parts. The first part aimed to identify the child’s age and assess the difficulties they encountered; the second part focus on investigating experiences of bullying, while the third part included two standardized scales, namely the Generalized Anxiety Disorder (GAD) and Major Depressive Disorder (MDD) sections of the Child and Adolescent Symptom Inventory-revision 4 (CASI-4) that Child and Adolescent Symptom Inventory-revision 4 (CASI-4), that report satisfactory psychometric data, including a test–retest reliability of *r* = 0.67 (*p* < 0.001) over a six-week period and an internal consistency of 0.74. The study indeed employed a variety of statistical methods, including both univariate and multivariate approaches, to examine the associations and factors related to emerging school refusal (SR) in autistic boys. Univariate methods such as Pearson and Spearman correlational analyses, logistic regression, Chi-square statistics, MANOVA, ANOVA, and Spearman correlations were utilized. These methods allowed for the exploration of relationships between emerging school refusal and various variables, including age, ASD-related characteristics, anxiety and depression scores, and the frequency of being bullied. The results revealed that boys with SR exhibited significantly higher GAD (*p* = 0.023; *η*2 = 0.097) and MDD (*p* = 0.013; *η*2 = 0.116) than boys without SR. Importantly, it was found that only the frequency of being bullied made a significant contribution to the emergence of SR (*p* = 0.004; exp(b) = 4.367). In the present study, over four-fifths of boys with autism reported experiencing bullying at school. Among those boys who reported being bullied, more than half approached their parents, requesting to abstain from returning to school the following day due to the bullying incidents. A noteworthy association was observed between the request to avoid school and the prevalence and frequency of bullying. Being bullied was a statistically significant contributor to emerging SR and was identify as a potential major ‘predictor’ of emerging SR among autistic boys.

A. J. McClemont et al. [29] conducted an evaluation of various factors influencing school refusal behavior attributed to bullying. These factors included diagnoses of Autism Spectrum Disorder (ASD) and Attention-Deficit/Hyperactivity Disorder (ADHD), child characteristics (behavioral problems and communication abilities), demographic variables, and school-related factors. The study comprised 97 parents and 154 children (age range 4–16). Specifically, children were grouped according to parent-reported diagnosis: ASD (*n* = 36), ADHD (*n* = 16); ASD + ADHD (*n* = 31), other diagnosis (anxiety disorders, mood disorders, disruptive behavior disorder, learning disorder, language/communication disorders, sensory/auditory processing disorder; *n* = 15), and no diagnosis (*n* = 56). Parents reported the frequency and relevance of their child’s school refusal behavior due to bullying by answering a few questions using the four-point Likert scale. Firstly, an Analysis of Variance (ANOVA) with Bonferroni correction was utilized to investigate the effect of diagnosis on lifetime school refusal due to bullying. For examining predictors of school refusal frequency, a multilevel model was initially considered due to the inclusion of siblings in the dataset. However, a model accounting for variance within families did not improve over the linear model, and an ordinal logistic regression was ultimately selected for parsimony. The model-building approach involved entering sets of predictors to test hypotheses, and model fit was assessed by comparing the − 2 log likelihood with the Chi-square statistic. The pseudo R2 statistic was used to interpret the percent variance explained by each model. Continuous variables, specifically child grade, were modeled, and assumptions regarding skewness, kurtosis, and collinearity were carefully considered. The results report that 35% of parents reported that their child has never refused to attend school due to bullying. There was a significant difference among these groups in terms of lifetime instances of school refusal behavior due to bullying (*p* < 0.001). Furthermore, the frequency of school refusal due to bullying was significant for youth with diagnoses of ADHD (odds ratio (OR): 3.54, 95% CI: 1.00, 12.56) and ASD + ADHD (OR: 4.85, 95% CI: 1.50, 15.71). In addition, children with both ASD and ADHD were more likely to have experienced school refusal behavior due to bullying (68%) compared to children with ASD (28%) or those without any diagnosis (18%) (*p* < 0.001). In conclusion, the highest rate of school refusal over the lifespan due to bullying was found in children with concurrent ASD + ADHD. Children with ASD + ADHD may be particularly vulnerable or have difficulty managing bullying victimization. Moreover, the high rates of comorbidity between ADHD and disruptive behavior disorders (i.e., oppositional defiant disorder, conduct disorder) suggest that children with ADHD in this sample may also engage in disruptive behaviors. Such behaviors are associated with bullying and indicate that behavioral problems increase the likelihood of school refusal due to bullying.

### School refusal behavior in children and adolescents with psychiatric disorders

#### Personality styles and functioning in adolescents

Carpentieri and colleagues [30] explored the differences in personality styles, adaptive functioning, and symptomatology between help-seeking adolescents with school refusal (SRa) and those who did not exhibit school refusal (non-SRa). The authors conducted a study involving 103 adolescent outpatients, with an average age of 16.2 (SD ± 1.14). Various scales and interviews were employed to characterize the study population, including the Hamilton Rating Scale for Anxiety (HAM-A) with a reliability index (Cronbach’s alpha) equal to 0.91, Hamilton Rating Scale for Depression (HAM-D) with a reliability index (Cronbach’s alpha) equal to 0.87, Young Mania Rating Scale (YMRS) with a reliability index (Cronbach’s alpha) equal to 0.79, Global Assessment of Functioning (GAF), Global Functioning Social Scale (GFSS), Global Functioning Role Scale (GFRS), and Shelder-Westen Assessment Procedure for Adolescents (SWAP-200-A) with a reliability index (Cronbach’s alpha) equal to > 0.80, with a median of 0.86. The study has undertaken several statistical analyses to explore associations and differences between groups related to School Refusal (SR), including socio-demographic variables and various measures of psychiatric symptoms. Although the term “multivariate analyses” is not explicitly mentioned, the study does employ a multivariable logistic regression to assess associations between several variables (GFSS, PD Schizotypal, PD Schizoid, HAM-A (tot), HAM-D (tot), and SR), thereby addressing potential confounding variables. The results revealed no significant differences between the two groups in terms of age, gender, school performance, parental education, annual financial income, or diagnosis. However, through independent Z-tests, it was observed that SRa individuals, in comparison to non-SRa individuals, exhibited higher levels of anxious and depressive symptoms (HAM-A *p* = 0.036; HAM-D *p* = 0.031), lower levels of global functioning (GAF, *p* < 0.001; GAF_past year, *p* = 0.025), as well as diminished levels of social and role functioning (GFSS, *p* = 0.003; GFRS, *p* = 0.002). SRa individuals also displayed higher rates of Schizoid (*p* = 0.046) and Schizotypal (*p* = 0.034) personality disorders. Furthermore, SRa individuals reported lower Health Functioning (*p* = 0.001) and a lower Q Health Index (*p* = 0.002). Finally, after the multivariable logistic regression model showed a significative effect only for GFSS (OR = 1.58, 95% CI: 1.00, 2.50, *p* = 0.05). SR has been shown to be closely linked to anxious and depressive symptomatology. Additionally, this study demonstrates that compromised adaptive functioning, particularly in the social domain, significantly increases the probability of SR. Regarding personality styles, it appears that emotional dysregulation, as well as self-criticism and a tendency toward self-inhibition, characterize individuals with SR to a greater extent.

#### Environmental factors

Al Keilani and Delvenne [8] investigated the role of environmental and family factors, as well as the influence of anxious and depressive symptoms on school refusal behavior. The study included a sample of 71 patients, ranging in age from 8 to 16 years, who were drawn from the Child and Adolescent Psychiatric Department of Queen Fabiola Children’s University Hospital. Clinical and demographic variables were assessed using a checklist developed by the authors, which included investigations about the patient’s gender, status, age of onset of school refusal behavior, age at assessment, duration of school refusal behavior, associated events, stressful life events, use of psychotropic medication, family psychiatric history, family composition, and individual psychiatric history. The study primarily relies on descriptive statistical analyses, providing means and standard deviations for the collected data. To compare samples, the study employs the T-student test and Mann-Whitney test. While these tests are useful for comparing means and distributions between two groups, the absence of explicit mention of multivariate analyses raises a concern about the potential control for confounding variables. The results revealed a significant gender difference: 70.42% of the participants with SR were male, compared to 29.57% who were female (*p* < 0.0003). School refusal behavior generally began in the first year (30.9%) or the second year (19.7%) of secondary school. Several risk factors were identified, including family separation (56.3%), conflicts at home (27%), contact rupture with the father (25.3%), maternal psychiatric illness (45.07%), paternal psychiatric illness (28.2%), academic difficulties (36.6%), and changing schools or moving homes (19.7%). Maltreatment was also prevalent in this population, with child abuse (30.9%), domestic violence (22.7%), child physical abuse (22.7%), neglect (27.2%), and child sexual abuse (27.2%) being reported. Regarding psychopathological diagnoses, anxiety (39.4%) and mood disorders (32.4%) were the most frequently observed conditions in the sample. A history of learning disabilities was noted in 30.56% of the inpatients. Finally, inpatients with SR exhibited a high frequency of relational difficulties with peers (48.57%). The results highlight that population exhibited more pronounced risk factors, including experiences of maltreatment, family separation or conflicts, and a parental history of psychiatric illness. Furthermore, school-related issues were prevalent in the cohort, with a quarter having experienced bullying and nearly half facing relational difficulties with peers. Additionally, one-third of patients had a history of learning disabilities.

#### Hikikomori syndrome in adolescents

Xavier Benarous et al. [31] aimed to determine the prevalence of Hikikomori syndrome (HKM) in a group of French adolescents exhibiting a severe form of social withdrawal and/or school refusal (SW/SR). They also aimed to understand how these subjects differed from other types of SW/SR. The study involved: 38 adolescents with school refusal without withdrawal from family or peer relations; 7 adolescents with school refusal and withdrawal from peer relations but not from family relations; 4 adolescents with intermittent SW/SR over the past three months; 18 adolescents with severe neurodevelopmental disorders, such as autism or intellectual disability, who were staying at home while awaiting placement in a special education facility. The study comprised a retrospective chart review of 191 French adolescents aged 12–18 years (Mean = 15.0, 44% boys) consecutively admitted to two inpatient units from January 2017 to December 2019. To assess the level of functioning and symptom severity, various tools were utilized, including the Children-Global Assessment of Functioning scale (C-GAF), Clinical Global Impressions-Severity scale (CGI-S), DEP-ADO, and a questionnaire to document substance use in the previous 12 months. Results revealed that 7% of participants with SW/SR (*n* = 83) met the criteria for Hikikomori syndrome (HKM) (*n* = 14, Mean age = 14.3, 64% boys), accounting for one in six adolescents with SW/SR. The study primarily relies on bivariate analyses to compare various factors among different groups. The comparisons are conducted using non-parametric tests like the Mann-Whitney test for continuous variables and the Fisher exact test for categorical variables. Effect sizes are calculated using Cramer’s Phi correlation coefficient for categorical variables and Cliff’s Delta score for the Mann-Whitney tests. Youths with HKM did not significantly differ from other forms of SW/SR in terms of demographic factors, academic performance, or psychosocial factors. Anxiety disorders (14/14–100%) were the most frequently associated diagnosis in HKM+ (Hikikomori Syndrome with social withdrawal and/or school refusal), followed by depressive disorders (9/14–64%). Among those with SW/SR, HKM + vs. HKM- patients had higher rates of anxiety disorders (*p* < 0.001; Odds Ratio, OR = 35.2; ɸeffect size = 0.51) and lower rates of disruptive behavioral disorders (OR = 0.03). None of the HKM + reported using illicit drugs or alcohol, compared to 25% of youths with other SW/SR. Participants with anxiety and depressive disorders who met HKM criteria (15% and 9%, respectively) showed a longer duration of symptoms, longer hospitalization, and a greater need for daily care facilities at discharge than those with Hikikomori syndrome. The study found that individuals with HKM and SW/SR shown higher rates of anxiety disorder and lower incidence of disruptive behavioral disorder. Additionally, these participants exhibited a prolonged duration of symptoms, extended hospitalization, and a greater need for daily care facilities at discharge compared to those without HKM. This discovery aligns with the perspective of HKM as a concept, distinct from psychiatric disorder categories but still clinically relevant in identifying a constellation of individual, familial, and cultural factors that influence the healthcare trajectories of adolescents with SW/SR.

## Discussion

As highlighted in the introduction, school refusal is a multifaceted issue influenced by various vulnerability factors. Our research indicates a link between school refusal and environmental, familiar, and individual risk factors, such as neurodevelopmental and psychiatric disorders. In particular, our study underscores the association between school refusal and exposure to familial stressors like poverty, parental unemployment, frequent relocations, parental neglect, domestic violence, and parental mental health issues. These findings are consistent with previous research indicating how challenging family and social circumstances contribute to school refusal [32–36] Additionally, the school environment and peer interactions play a role in school refusal, with factors like bullying and socialization difficulties contributing to the phenomenon [37, 38].

Our findings referring to individual risk factor align also with prior studies that have extensively explored the strong correlation between school refusal and psychiatric disorders [32, 35] Notably, children with school refusal exhibited a threefold increase in psychiatric disorders compared to their peers without attendance issues. Particularly, they faced elevated risks of Social Anxiety Disorder and depression [39].

The goal of this narrative review was to contribute to the updating of recent findings about the clinical significance of school refusal behavior, its negative impact on psychological well-being of children and adolescents and its relationship with the most common psychopathological conditions during development.

### School refusal behavior: analysis of risk factors and impacts on student well-being

School refusal behavior can be associated with various emotional and behavioral conditions that may serve as risk factors, such as a low self-concept, cyberbullying, specific affective profiles, excessive technology use, and psychopathological vulnerabilities.

Regarding the self-concept, in our narrative review, an interesting result emerges from a study of Gonzalvez et al [24]hat showed that a negative self-perception regarding competence in academic field and social relationship could affect the presence of school refusal behavior in adolescents that showed a higher level of dissatisfaction and a lower perception of their emotional well-being. This finding is consistent with the results of other studies [40, 41] finding that a negative self-concept is implicated in the development and maintenance of school refusal behavior. In the scholastic context, children or adolescent must be able to cope with numerous “task”, such as obtaining adequate performance on academic tests, relating daily with their classmates and teachers showing participation in lessons and social initiative. If thoughts and feelings about oneself in the scholastic effect are negative, the risk that the child or adolescent manifests a tendency to avoidance or, in severe cases, school refusal behavior, is higher. By contrast, having a positive self-concept and good relationships with peers or teachers can prove to be a protective factor against school attendance problems. Overall, self-concept, self-esteem and self-efficacy become an important affective-motivational variable in the explanation of school refusal behavior. However, other psychological risk factors have also been studied as implicated in school refusal behavior. These are in relationship with environmental risk factors such cyberbullying. For example, Delgado et al. [26] identified school refusal behavior as an avoidance strategy of negative emotions and social or assessment situations in 38% of students. Moreover, students who refused to attend school exhibited lower social skills and used technology as a measure of socialization with their peers. Technology has undoubtedly transformed social interactions, and while it offers various opportunities for communication, it may contribute to social isolation and vulnerability to cyberbullying. Indeed, in this study a significant association emerged between adolescents with high levels of school refusal behavior and exposure to cyberbullying. This finding has been supported by previous studies, where cyber-victims avoided school because they did not feel safe [42]. . Consequently, school avoidance served as a strategy to not meet face-to-face with their aggressors and attempting to reduce the fear and anxiety they experienced. However, according to Fujita et al. [25], 40% of adolescents with school refusal behavior exhibit excessive time spent on the internet. In the same study, adolescents with school refusal behavior and problematic use of internet showed higher depressive symptoms, higher anxiety, and higher difficulties in social, academic, and family daily functioning compared with adolescents with school refusal behavior without problematic use of internet. These findings support the idea that problematic use of internet in adolescents with school refusal behavior could be a potential risk factor for mental disorders in adolescence. Regarding the specific affective profiles, in a subsequent study, Gonzavez et al. [24] found that a “self-destructive profile” have a higher disposition for school refusal behavior. The term “self-destructive” implies a pattern of emotional and behavioral tendencies, such us fear, anger, nervousness, lack of interest, guilt, shame and, above all, high levels of avoidance towards social stimuli and situations perceived as aversive and eliciting negative affectivity. Therefore, these children and adolescents may have a tendency to experience the school context as stressful and the avoidance of this through school refusal behavior might be indicative of difficulties in coping with social challenges that potentially isolate them from social interactions.

All these aspects have shown a relationship with anxious or depressive symptoms and anxiety and depression disorder [43]. Other authors have also found similar results, such as Sanmartin et al. [44], that in a previous study, have found a relationship between self-destructive profile, school refusal behavior and higher levels of social anxiety.

### School refusal behavior in children and adolescents with neurodevelopmental disorders

Regarding the clinical significance of school refusal behavior in children and adolescents with neurodevelopmental disorders, all three studies included showed a specific profile of emotional, neuropsychological and social functioning within this clinical population. Regarding emotional aspects, in Munkhaugen et al. [27] was observed that children and adolescents with autism spectrum disorder (ASD) and school refusal behavior (SRB) exhibit more internalizing problems than those SRB. Specifically, these problems consist in withdrawn and depressive symptoms, followed by anxiety symptoms and somatic complaints. These finding are in line with previous studies conducted by Havik et al. [45] and Ingles et al. [46] in the general child and adolescent population which showed that anxiety, depression and somatic complaints were the most prevalent symptoms associated to SRB. The relationship between internalizing problems and SRB in children and adolescents with ASD needs to be further explored with experimental study design that include more information about the school environment, clinical history with psychiatric comorbidities associated with ASD, traumatic experience school-related and poor mentalizing skills. However, we may speculate that, as highlighted in previous studies [47, 48] in general child and adolescent population, low expectations in coping with stressful situations in school and negative automatic thoughts about relationships with peers and teachers could be associated to SRB in children and adolescents with SRB. Consequently, school could be represented in the minds of these children as a threatening context with rejection as a behavioral response.

Regarding neuropsychological aspects, ASD children and adolescents with school refusal behavior displayed higher rates of executive function deficits [27] than those without SRB. Specifically, impaired ability to initiate the activities followed by planning/organizing and shifting difficulties. No differences were found in capacities to inhibit and monitor behavior or in working memory. These results are in line with Ohmann et al. [49]. In addition, lack of initiative and impaired shifting and planning were strongly associated with problems in overall adaptive, social and school functioning in children and adolescents with ASD [50, 51]. Further studies are needed to examine the relationship between executive dysfunction and SRB. Despite this, we could hypothesize that, in children and adolescents with ASD and SRB, the initiation deficits, together with planning/organizing difficulties, could limit the ability to ask for information and support from teachers and peers necessary for them to start tasks and social activities. Teachers and peers could interpret this as lack of interest resulting in negative feedback for the children and adolescents with ASD. Consequently, school could be represented in the minds of these children as a threatening context with school refusal as a behavioral response.

Regarding social aspects, in our review, ASD children and adolescents with SRB showed lower social motivation compared to those who did not refuse school. Importantly, this lower social motivation appeared to be independent of impaired social skills and the ability to recognize socially relevant cues typically associated with ASD. To date, we have not identified studies on characteristics within the social domain associated to SRB in this clinical population. Despite this, we propose that this lower social motivation could be attributed to temperamental and personality traits (e.g., introversion) or previous negative experiences related to social interactions (e.g., bullying).

About this, the studies included in our review also showed, in ASD children and adolescents with SRB, a relationship between being bullied and experiencing school refusal behavior. Specifically, as highlighted by Bitsika et al. [28], being bullied explains school refusal behavior more than ASD-related difficulties, anxiety and depression symptoms. Indeed, while ASD children and adolescents with SRB showed higher levels of anxiety and depression compared to those without SRB, the frequency of being bullied is a stronger “predictor” of school refusal behavior among ASD children and adolescents. The reason why bullying is linked to school refusal behavior in ASD is not yet known. However, there was no significant association between the difficulties typically related to the major feature of ASD (e.g. socializing and communicating difficulty), and emerging SRB. Nevertheless, there was a significant association between their tendency to externalizing behaviors (e.g. restricted and repetitive behaviors) and the frequency being bullied. This is partially supported by McClemont et al. [29], where 35% of parents of children with ASD, ADHD, ASD + ADHD and other diagnoses, report that their children manifested school refusal behavior due to bullying. Specifically, children with ASD + ADHD seem to be more vulnerable to bullying, probably due to the manifestation of externalizing behaviors. Overall, these findings suggests that behavioral problems increase the likelihood of being bullied and, as a result, they lead to school refusal behavior.

### School Refusal Behavior in children and adolescents with psychiatric disorders

In our review, some studies investigated profile of children and adolescents with school refusal behavior in terms of relationship with psychiatric problems. For example, Carpentieri’s [30] research examined school refusal behavior and its association with psychiatric symptoms, particularly anxious and depressive symptoms, alongside compromised adaptive and social functioning. Similarly, in Al Keilani and Delvenne [8], patients with school refusal behavior were diagnosed with different internalizing disorder, primarily anxiety (50%) and to a lesser extent, depression. These findings are in line with earlier studies that have indicated a link between school refusal behavior and lower emotional stability, as well as difficulties in interpersonal relationships [52, 53]. The presence of these psychiatric symptoms emphasizes the urgency to address school refusal behavior comprehensively. Indeed, during adolescence, school refusal behavior should also be examined in relation to specific personality traits that emerge in this period of development. Regarding this, Carpentieri et al. [30] investigated the association between school refusal behavior and specific personality traits. The results demonstrated that adolescents with school refusal behavior exhibited higher schizoid and schizotypal characteristics, predisposing them to displaying avoidant personality traits. Similarly, Lounsbury et al. [54] have highlighted the correlation between school refusal behavior and personality traits such as low agreeableness and introversion, which contribute to interpersonal difficulties and reduced enjoyment in social settings. Overall, these findings indicate that certain personality traits may play a crucial role in the development and perpetuation of school refusal behavior. Further, deepening into these findings, it has been found that inhibited and self-critical personality style is closely related to internalizing psychopathology and includes feelings of embarrassment and shame in social contexts, high standards, perfectionistic tendencies, and a tendency toward self-criticism. Adolescents with these personality styles and internalizing symptoms tend to more frequently avoid feelings of frustration from social and performance challenges. The presence of such emotional patterns, along with problematic emotional dysregulation, may contribute to the emergence of school refusal behavior. However, Al Keilani and Delvenne [8]also highlighted the multidimensionality of the nature of school refusal behavior among adolescents, identifying potential risk factors. First, their observation of a sex difference in favor of males in inpatients with school refusal behavior brings attention to potential gender-specific factors influencing school avoidance behaviors. Second, they identified transitional periods between different school cycles, such as entering elementary school, middle school, and high school, as crucial periods that may induce heightened stress and anxiety, leading to increased rates of school refusal behavior. Third, exposure to maltreatment (domestic violence, physical abuse, neglect, and sexual abuse), family separations, maternal psychiatric illnesses, and academic problems like learning difficulties could be potential risk factors for school refusal behavior. Finally, consistent with earlier research findings [25, 26], they also observed that 27.94% of the patients reported being victims of bullying, while 48.7% experienced difficulties in peer relationships. These results further underscore that adolescents with school refusal behavior manifest obstacles in social integration, which may arise from restricted social interactions, shyness, and difficult relationships [39]. The relationship between difficult relationship and school refusal behavior was examined by Benarous et al. [31]in a sample of adolescents inpatients with school and social behavior associated with hikikomori syndrome (7%) defined as a chronic and severe form of social withdrawal. This sample exhibited a long duration of school and social refusal and low level of psychiatrics symptoms, such as anxiety and depression disorders, during the hospitalization. However, when faced with situations that frightened them, such as being allowed to return to school, these patients manifested their psychiatric symptoms. The authors also observed an intense need for perfectionism and control in academic domains and in peer relations among these inpatients. This observation indicates a potential connection between their maladaptive coping mechanisms and the perpetuation of their withdrawal behaviors. The pursuit of perfectionism and rigid control may serve to avoid potential failures on social challenges, reinforcing their reluctance to engage in school and social activities experienced by them as stressful.

### Strengths and limitations

This review is the first where clinical significance is explored in relation to both neurodevelopmental disorders and psychiatric disorders. However, some limitations may be considered. In conducting our research, we took significant steps to ensure the reliability and quality of our review. One key aspect of this was conducting a thorough bias analysis. While most of the studies included in our review were found to have a low risk of bias across all domains (selection bias, performance bias, detection bias, attrition bias, and reporting bias), it’s important to note that two studies were classified as having a medium risk of bias overall. This suggests that there may be limitations in the study design or execution that could impact the reliability of their results.

Another consideration is the variability among the included studies. However, despite our efforts, there are limitations regarding participant selection and sampling methods that may affect the generalizability of our findings. For instance, participants were not consistently drawn from clinical populations, which could limit the applicability of our results to broader groups.

Furthermore, the geographic focus of the included studies on populations from Europe and North America is another potential limitation. This could restrict the generalizability of our findings to other cultural contexts, as the lack of cross-cultural representation may limit the applicability of our results to diverse populations.

Overall, while we have taken rigorous steps to ensure the reliability and quality of our review, it’s essential to recognize and address these limitations when interpreting and generalizing our findings. Future research efforts could incorporate different populations, standardized methodologies, and broader geographic representation, thereby enhancing the robustness and applicability of future studies in this area.

## Conclusions

The present narrative review synthesized the recent literature on clinical significance of school refusal behavior, its negative impact on psychological well-being of children and adolescents and its relationship with the most common psychopathological conditions during childhood and adolescence. Our results suggest the heterogeneity of school refusal behavior and its relationship with different vulnerability factors (e.g. temperamental traits, difficult relationship, bullying) and different psychopathological condition (e.g. neurodevelopmental disorders, psychiatric disorders). Psychiatrists and Psychologists should be aware of this when assess children and adolescents with school refusal behavior and plan patient-tailored therapeutic interventions. For example, children and adolescents with school refusal behavior may be supported with a Cognitive Behavioral Therapy approach, to enhance recognition of cognitive biases about school as a threatening and stressful context, the regulation of negative emotions associated with school attendance (e.g. anxiety, depressive symptoms). Finally, therapeutic intervention programs (e.g. social skills training, problem-solving training) conducted directly in the school context could improve the psychological well-being of children and adolescents at school, increase their coping capacity with respect to academic performance and social relationships and, ultimately, prevent school refusal behavior. Future studies are needed. Longitudinal future studies should be conducted, collecting information on the onset and clinical course of school refusal behavior. In these studies, the role of parental psychological distress should be evaluated in the onset and maintenance of school refusal behavior.

### Clinical implications

A relevant issue in the area of developmental psychopathology concerns the accurate recognition of signals related to school refusal and any underlying anxiety disorders. Children often have difficulty expressing their emotions, which is why they tend to report somatic symptoms such as stomachaches, headaches, nausea, and chest pain. These symptoms then become a reason for consultation with the pediatrician, who has the valuable task of promoting early diagnostic framing and understanding whether the reported issue is psychopathological or medical in nature. This decision must be made based on the presence and duration of the reported symptoms and how much they compromise the girl’s normal daily activities (e.g., school attendance, completion of tasks, participation in sports, relationships with friends and family, etc.). It is also necessary to exclude any physiological causes through the prescription of screening tests (e.g., blood tests, ECG, etc.). To make an initial diagnostic assessment, the pediatrician may ask direct questions about possible behavioral changes that have occurred recently, such as a decrease in academic performance or changes in eating or sleep habits. It is also useful to investigate difficulties in school attendance, discomfort not attributable to organic issues that are more pronounced at school entry, during homework, or during group activities. In Table 3, we have summarized the possible emotional, cognitive, and behavioral changes that children and adolescents may manifest, which should alert parents, teachers, and the referring pediatrician.

**Table 3:** Emotional, cognitive, and behavioral changes

Emotional, cognitive, and behavioral changes in children/adolescents that should alert parents and clinicians [55]	
-Inability to cope with daily activities as usual. -Changes in sleep patterns and/or eating habits; -Excessive complaints about physical discomfort; -Disregard for authority, skipping school, theft, or damaging others’ property; -Intense fear of gaining weight; -Long-lasting negative moods, often accompanied by poor appetite and thoughts of death; -Substance and/or alcohol abuse; -Frequent fits of rage; -Changes in academic performance, with low grades despite their efforts;	-Loss of interest in activities and the company of friends who usually bring joy; -Significant increase in time spent alone; -Excessive worry or anxiety; -Hyperactivity; -Persistent nightmares or night terrors; -Persistent disobedience or aggressive behavior; -Frequent outbursts of anger; -Hearing voices or seeing things that aren’t there (hallucinations).

In case of a referral to specialized figures such as a psychologist or child neuropsychiatrist, it is useful for the pediatrician to establish collaboration with the specialist. The pediatrician is also required to gather information and stay updated on the distress presented by the child in order to implement a first psychoeducational intervention aimed at explaining to the child and parents the difficulty presented and the importance of specialist interventions such as cognitive-behavioral psychotherapy and possible pharmacological treatment. The pediatrician can also recommend books that facilitate the family’s understanding of the distress and the implementation of self-help strategies to provide advice on modifying some habits, such as regulating sleep, the time spent on electronic devices exposing children and adolescents to increased levels of anxiety and stress. In light of the above, we can affirm that the pediatrician plays a fundamental role in prevention, health education, and the overall promotion of the health of children and adolescents.

## Data Availability

Not applicable.

## Abbreviations

ADHD: Attention Deficit and Hyperactivity Disorder
ANE: Avoidance of Negative Affectivity
ASD: Autism Spectrum Disorder
BRIEF: Behavior Inventory of Executive Function
CASI-4: Child and Adolescent Symptom Inventory-revision 4
CBCL: Child Behavior Checklist
C-GAF: Children-Global Assessment of Functioning scale
CGI-S: Clinical Global Impressions-Severity scale
DSM: Diagnostic and Statistical Manual of Mental Disorders
ESE: Escape from social and evaluative situations
GAD: Generalized Anxiety Disorder
GAD-7: General Anxiety Disorder-7
GAF: Global Assessment of Functioning
GEC: Global Executive Composite
GFRS: Global Functioning Role Scale
GFSS: Global Functioning Social Scale
HAM-A: Hamilton Rating Scale for Anxiety
HAM-D: Hamilton Rating Scale for Depression
HKM: Hikikomori syndrome
HRQOL: health-related quality of life
IAT: Internet Addiction Test
ID: Intellectual Disability
MDD: Major Depressive Disorder
PA: Pursuit of Attention
PANAS-C-SF: Positive and Negative Affect Schedule Short Form
PHQ-9: Patient Health Questionnaire-9
PIU: Problematic Internet Use
PRISMA: Preferred Reporting Items for Systematic Reviews and Meta-Analyses
PTR: Pursuit of Tangible Reinforcement
QCD: Questionnaire-Children with Difficulties
SAP: School Attendance Problem
SDQ: Self-Description Questionnaire
SPH: Screening of Harassment Among Peers
SR: School Refusal
SRAS-R: School Refusal Assessment Scale-Revised
SRAS-R-C: School Refusal Assessment Scale-Revised for Children
SRB: School Refusal Behavior
SRS: Social Responsiveness Scale
SW: social withdrawal
SWAP-200-A: Shelder-Westen Assessment Procedure for Adolescents
YMRS: Young Mania Rating Scale
